# Menopausal Hormone Therapy and Cardiovascular Risk: Current Evidence and Clinical Implications

**DOI:** 10.3390/medsci14020298

**Published:** 2026-06-10

**Authors:** Catalin M. Buzduga, Amelian M. Bobu, Roxana Covali, Claudia Florida Costea, Andrei I. Cucu, Mariana Graur, Emilia Patrascanu, Iustina Solomon-Condriuc, Alexandru Carauleanu

**Affiliations:** 1 Faculty of Medicine, Grigore T. Popa University of Medicine and Pharmacy Iasi, 700115 Iasi, Romania; catalin.buzduga@umfiasi.ro (C.M.B.); ana.covali@umfiasi.ro (R.C.); patrascanu.emilia@umfiasi.ro (E.P.);; 2 Faculty of Medicine and Biological Sciences, Stefan cel Mare University of Suceava, 720229 Suceava, Romania; andrei.cucu@usm.ro (A.I.C.);

**Keywords:** menopausal hormone therapy, cardiovascular disease, coronary heart disease, venous thromboembolism, timing hypothesis

## Abstract

Background: Menopausal hormone therapy (MHT) effectively relieves vasomotor symptoms, but its cardiovascular safety remains influenced by timing, formulation, and route of administration. Methods: This narrative review summarizes evidence from major randomized trials (WHI, HERS, ELITE, DOPS) and observational studies, along with mechanistic data on the vascular and metabolic effects of MHT. Results: Although early studies suggested cardioprotection, randomized trials showed no cardiovascular benefit, and in some cases, increased risks of coronary events, stroke, and venous thromboembolism, particularly in older women or those with established cardiovascular disease. The “timing hypothesis” indicates that early initiation after menopause may have neutral or modestly favorable effects, whereas late initiation is associated with adversity. Oral estrogen is linked to higher thromboembolic and stroke risk compared with transdermal formulations. Evidence on atrial fibrillation and heart failure remains limited. Conclusions: MHT should not be used for cardiovascular disease prevention. Current evidence suggests that younger women in the early postmenopausal period may derive the greatest benefit with the lowest risk from individualized hormone therapy regimens, particularly those using transdermal estrogen. Treatment decisions should be guided by careful cardiovascular risk assessment and targeted to symptom relief and osteoporosis prevention.

## 1. Introduction

Menopause represents a major physiological transition in a woman’s life, clinically defined as the absence of menstruation for at least 12 consecutive months in the absence of other physiological or pathological causes [1,2]. The term “menopause” derives from the Greek words *men* (month) and *pausis* (cessation) and describes the permanent discontinuation of menstrual cycles [3]. It most commonly occurs between the ages of 50 and 52 years, with approximately 95% of women experiencing their final menstrual period between 44 and 56 years of age [4]. This stage is characterized by a decline in ovarian hormone production, particularly estradiol and progesterone, accompanied by an increase in follicle-stimulating hormone (FSH) levels. It is also associated with a significant rise in cardiovascular risk, making menopause a critical window for the evaluation of preventive strategies, including menopausal hormone therapy [2].

During the menopausal transition, a series of endocrine changes occur, regulated by the hypothalamic–pituitary–ovarian axis. Reduced ovarian production of inhibin leads to increased follicle-stimulating hormone (FSH) levels, followed by fluctuations and a subsequent decline in estradiol concentrations [5]. These changes characterize the perimenopausal period, a transitional stage toward menopause associated with irregular menstrual cycles and frequent vasomotor symptoms, such as hot flashes and night sweats [2].

Beyond clinical manifestations, estrogen deficiency affects endothelial function, lipid metabolism, and inflammatory pathways, contributing to the increased cardiovascular risk observed after menopause [5,6,7]. Vasomotor symptoms during the menopausal transition have also been associated with elevated cardiovascular risk, suggesting a link between the clinical manifestations of menopause and the development of cardiovascular disease [8]. The decline in estrogen levels after menopause has been associated with a substantial increase in cardiovascular risk, as demonstrated in the Framingham Study, while early estrogen deficiency, including premature or surgically induced menopause, further amplifies the risk of coronary artery disease, underscoring the need for preventive strategies [9,10,11].

Hormone replacement therapy (HRT), also referred to as menopausal hormone therapy (MHT), represents the most effective option for alleviating vasomotor symptoms and the genitourinary syndrome of menopause, while also contributing to the prevention of bone loss [2,12,13]. The therapeutic regimen depends on uterine status: women with an intact uterus require the addition of a progestogen for endometrial protection, whereas hysterectomized women may receive estrogen monotherapy [14]. MHT can be administered via multiple routes, including oral, transdermal, or vaginal, allowing for individualized treatment approaches [15,16].

Initially, estrogens were used as monotherapy; however, progestogens were subsequently introduced to reduce the risk of endometrial hyperplasia and carcinoma associated with unopposed estrogen exposure in women with an intact uterus [17]. In the 1990s, hormone therapy was widely used for the management of menopausal symptoms and for its presumed benefits in the prevention of osteoporosis and cardiovascular disease. However, the publication of randomized trial results from the Heart and Estrogen/Progestin Replacement Study (HERS) [18] and the Women’s Health Initiative (WHI) [19,20] demonstrated an increased cardiovascular risk associated with certain therapeutic regimens, leading to a marked decline in hormone therapy use.

The use of MHT has declined significantly worldwide over the past two decades, particularly following the publication of the initial results of the Women’s Health Initiative. In the United States, the prevalence of MHT use among postmenopausal women decreased from approximately 26.9% in 1999 to 4.7% in 2020 [21]. In Europe, usage varied considerably between countries in the early 2000s, ranging from less than 5% in some Southern European countries to over 25% in Nordic regions; following the WHI and HERS studies, reductions of 50–77% were observed, with prevalence falling below 10% in most countries by 2010 [22].

In Asia, MHT use remains relatively low, with data from the Asian Menopause Survey indicating a prevalence of 19% for prior use and 7% for current use [23,24]. In Australia, after the initial decline in the early 2000s, usage appears to have stabilized at a moderate level, with a prevalence of approximately 10–13% in the general postmenopausal population and up to 22.5% among symptomatic women [25,26]. In contrast, in Africa, available data suggest minimal or absent use of MHT, with some studies reporting no use at all in certain populations [27].

This narrative review synthesizes current evidence on the effects of menopausal hormone therapy (MHT) on cardiovascular risk, integrating data from landmark randomized clinical trials, contemporary observational studies, and current recommendations from major scientific societies. It examines the impact of different therapeutic approaches on vascular function and cardiovascular risk profiles, as well as the influence of treatment timing, hormone formulation, and route of administration. By combining evidence from classical and emerging studies, including recent data on arrhythmias and heart failure, this review aims to provide an updated and clinically relevant perspective on the role of MHT in contemporary cardiovascular risk assessment and management.

## 2. Methodology

This narrative review provides a focused synthesis of the available evidence regarding the impact of menopausal hormone therapy (MHT) on cardiovascular risk in postmenopausal women. A targeted literature search was conducted using PubMed and Google Scholar, employing combinations of the terms “menopausal hormone therapy”, “hormone replacement therapy”, “menopause”, “cardiovascular disease”, “coronary heart disease”, “stroke”, “venous thromboembolism”, “heart failure”, “atrial fibrillation”, “estrogen”, “progestin”, and “risk factors”.

The search covered publications from 1998 through March 2026, corresponding to the period during which the most influential clinical studies evaluating the cardiovascular effects of MHT were published. Both landmark and contemporary studies were considered to provide a comprehensive overview of the evolving evidence base.

Studies were selected based on their clinical relevance, methodological quality, and contribution to the understanding of cardiovascular outcomes associated with MHT. Particular emphasis was placed on major randomized clinical trials, large cohort studies, and contemporary position statements and guidelines issued by leading scientific societies. Priority was given to investigations reporting clinically relevant cardiovascular outcomes, including coronary heart disease, myocardial infarction, stroke, venous thromboembolism, heart failure, and atrial fibrillation, as well as studies evaluating the influence of treatment timing, hormone formulation, and route of administration.

As this work was designed as a narrative review, the literature was synthesized qualitatively rather than through a formal systematic review process. When conflicting findings were identified, greater consideration was given to large-scale studies, long-term follow-up data, and guideline-supported evidence.

## 3. Mechanisms of Cardiovascular Effects of Menopausal Hormone Therapy

Understanding the mechanisms through which menopausal hormone therapy (MHT) influences the cardiovascular system is essential for optimizing therapeutic strategies and individualizing treatment. Estrogens and progesterone exert pleiotropic effects on the cardiovascular system, involving vascular, metabolic, and electrophysiological pathways. Although endogenous estrogens are believed to exert cardioprotective effects through favorable effects on lipid metabolism, endothelial function, vascular reactivity, and inflammatory pathways, these physiological benefits have not been consistently reproduced with menopausal hormone therapy in randomized clinical trials [28,29,30]. Large studies, including the Heart and Estrogen/Progestin Replacement Study (HERS) and the Women’s Health Initiative (WHI), failed to demonstrate overall cardiovascular benefit, and in certain populations, reported an increased risk of adverse cardiovascular events [18,19,31,32,33].

Hormone therapy affects the cardiovascular system through well-defined vascular, electrical, and metabolic mechanisms. Estrogen increases nitric oxide bioavailability and promotes endothelium-dependent vasodilation; however, prolonged exposure may alter microvascular responses by shifting toward hydrogen peroxide-mediated pathways. In parallel, progesterone induces direct vasorelaxation by inhibiting L-type calcium channels and reducing Ca^2+^ influx in vascular smooth muscle [33,34,35,36,37,38,39].

At the cardiac level, these hormones modulate remodeling by reducing fibrosis and inflammation, maintaining electrical conduction, and regulating calcium homeostasis, but may also influence ventricular repolarization, with potential proarrhythmic effects in certain contexts [40,41,42]. Metabolically, hormone therapy reduces the total cholesterol, LDL-C, apolipoprotein B, and lipoprotein(a), improves the atherogenic ratios and insulin sensitivity, but may slightly increase the triglyceride levels, resulting in an overall effect that depends on the individual risk profile [43,44,45].

## 4. Clinical Evidence

The effects of menopausal hormone therapy (MHT) on the cardiovascular system have been extensively evaluated in major clinical trials that have significantly influenced medical guidelines. Understanding how this body of evidence has evolved over time is essential for the accurate interpretation of current recommendations. Currently, medical societies do not recommend hormone therapy for the primary or secondary prevention of cardiovascular disease. Nevertheless, subsequent analyses have suggested that the timing of treatment initiation may influence the benefit–risk profile, with initiation closer to the onset of menopause being associated with more favorable outcomes [31].

Current recommendations from major professional societies are summarized in Table 1.

### 4.1. Contemporary Swedish Nationwide Trial

A recent Swedish nationwide emulated target trial including 919,614 women aged 50–58 years evaluated the cardiovascular safety of contemporary MHT regimens. Oral estrogen–progestin therapy was associated with increased risks of ischemic heart disease (HR 1.21, 95% CI 1.00–1.46) and venous thromboembolism (HR 1.61, 95% CI 1.35–1.92), whereas transdermal estrogen preparations showed a more favorable cardiovascular safety profile. Tibolone was associated with increased risks of overall cardiovascular disease (HR 1.52, 95% CI 1.11–2.08), myocardial infarction (HR 1.94, 95% CI 1.01–3.73), and cerebral infarction (HR 1.97, 95% CI 1.02–3.78) [50].

### 4.2. Early Versus Late Intervention Trial with Estradiol (ELITE)

ELITE was a randomized, double-blind, placebo-controlled study designed to test the hypothesis that the vascular effects of hormone therapy vary according to the timing of initiation. The effect of estradiol on the progression of carotid intima-media thickness (CIMT) differed significantly between women in early versus late postmenopause (*p* for interaction = 0.007), with a reduction in the progression of subclinical atherosclerosis observed only in the early postmenopause group [30].

These findings support the timing hypothesis; however, interpretation should consider baseline differences between groups that reflect not only time since menopause but also underlying vascular risk profiles. An important limitation is the use of a surrogate marker rather than clinical cardiovascular outcomes. Additionally, coronary parameters assessed by computed tomography did not differ significantly between groups [30].

Therefore, ELITE provides important mechanistic evidence supporting the early initiation of hormone therapy, although its direct clinical relevance remains limited.

### 4.3. Danish Osteoporosis Prevention Study (DOPS)

The Danish Osteoporosis Prevention Study (DOPS) was a randomized, open-label trial with blinded endpoint assessment (PROBE design) that investigated the long-term effects of hormone therapy initiated early after menopause. After 10 years of treatment and up to 16 years of follow-up, HRT significantly reduced the risk of the composite endpoint (all-cause mortality, myocardial infarction, or heart failure) (hazard ratio [HR], 0.48; 95% CI, 0.26–0.87; *p* = 0.015), with the effect persisting in extended analyses (HR, 0.61; 95% CI, 0.39–0.94). No significant increases were observed in the risk of cancer (HR, 0.92), stroke (HR, 0.77), or venous thromboembolism. Interpretation of these findings is limited by the open-label design and the relatively small sample size [51]. These results support the timing hypothesis, suggesting that the early initiation of HRT may confer cardiovascular benefit.

### 4.4. Kronos Early Estrogen Prevention Study (KEEPS)

KEEPS was a multicenter, randomized, double-blind, placebo-controlled trial designed to evaluate whether the early initiation of hormone therapy could slow the progression of subclinical atherosclerosis in recently postmenopausal women without preexisting cardiovascular disease. The study was motivated by discrepancies between observational data and findings from WHI and HERS, as well as by the hypothesis of a “window of opportunity” for the beneficial vascular effects of estrogen [52,53].

A key strength of KEEPS lies in its inclusion of a younger population closer to menopause onset compared with prior trials, and in its use of lower doses and formulations more reflective of contemporary clinical practice, including transdermal estradiol and micronized progesterone. However, the strict selection criteria, which excluded women with higher cardiovascular risk or significant coronary artery calcification, limit the generalizability of the findings.

Another important limitation is the reliance on surrogate markers of atherosclerosis, particularly CIMT and coronary artery calcium score, rather than clinical cardiovascular events. Accordingly, KEEPS was designed primarily to test a mechanistic hypothesis rather than to demonstrate direct clinical benefit [52,53].

Overall, KEEPS played an important role in reshaping the conceptual framework of hormone therapy, supporting the notion that age, timing of initiation, and formulation may influence cardiovascular outcomes.

### 4.5. Women’s International Study of Long Duration Estrogen After Menopause (WISDOM Study)

WISDOM study was a randomized, double-blind, placebo-controlled trial designed to evaluate the long-term risks and benefits of hormone therapy in postmenopausal women. Although the study was terminated prematurely, available data showed an increased risk of major cardiovascular events in the combined therapy group compared with the placebo (7 vs. 0 events; *p*= 0.016), as well as a markedly elevated risk of venous thromboembolism (HR: 7.36; 95% CI, 2.20–24.60). No significant differences were observed for cerebrovascular events, cancer, or all-cause mortality [54].

Interpretation of these findings should consider the relatively advanced mean age of participants (approximately 63 years), reflecting the initiation of therapy long after menopause, which limits applicability to women starting hormone therapy earlier. In addition, early trial termination and short follow-up duration reduce the ability to assess long-term outcomes. Nevertheless, the WISDOM results are consistent with those of WHI and support the lack of cardiovascular benefit of hormone therapy when initiated late after menopause.

### 4.6. Women’s Health Initiative (WHI)

The Women’s Health Initiative (WHI) was a randomized, double-blind, primary prevention trial initiated in 1993 to evaluate the effects of hormone therapy on chronic disease outcomes in postmenopausal women. The study included two arms: combined estrogen–progestin therapy and estrogen-only therapy [20,55].

The combined therapy arm was terminated prematurely in 2002 after a mean follow-up of 5.2 years due to an unfavorable risk–benefit profile, characterized by an increased risk of cardiovascular and neoplastic events [55]. In contrast, the estrogen-only arm continued until 2004 and demonstrated a more neutral cardiovascular profile, without a significant increase in the risk of coronary heart disease [20].

Overall, the results showed an increased risk of coronary heart disease, stroke, and venous thromboembolism, with no effect on all-cause mortality. Although the absolute risks were small, they were clinically meaningful at the population level.

Interpretation of these findings is influenced by the heterogeneity of the study population, particularly with respect to age and time since menopause. Most participants initiated therapy long after menopause, in a context of already elevated cardiovascular risk. Subsequent subgroup analyses suggested that younger women or those closer to menopause onset may have a different risk profile, leading to the formulation of the “timing hypothesis” [55]. Several important limitations should be acknowledged, including the evaluation of a single therapeutic regimen, high rates of nonadherence, and early trial termination, all of which limit the assessment of long-term effects. Further analyses within WHI demonstrated substantial heterogeneity in cardiovascular outcomes according to age, presence of vasomotor symptoms, and time since menopause. These findings suggest that the risk-benefit profile of hormone therapy is not uniform across populations, with trends toward lower—or potentially favorable—risk among younger women and those in early postmenopause compared with those initiating therapy later. These observations underpinned the development of the “window of opportunity” hypothesis [56].

Overall, WHI remains the landmark study demonstrating that combined hormone therapy is not appropriate for the primary prevention of cardiovascular disease, while also highlighting the critical importance of timing of initiation. These findings may, at least in part, explain the discrepancies observed among subsequent clinical trials, which are discussed in the following section.

### 4.7. Heart and Estrogen/Progestin Replacement Study (HERS)

HERS was a randomized, double-blind, placebo-controlled secondary prevention trial evaluating the effects of combined estrogen–progestin therapy in postmenopausal women with established coronary artery disease. The intervention did not reduce the overall risk of coronary events (hazard ratio [HR], 0.99; 95% CI, 0.80–1.22) [18].

Time-dependent analysis revealed a nonuniform pattern of effect, with an increased risk during the first year (HR, 1.52) followed by a trend toward risk reduction in subsequent years (HR, 0.67 in years 4–5; *p* for trend = 0.009). These findings suggest a possible early adverse effect followed by a delayed benefit; however, interpretation is limited by the loss of randomization inherent to interval-based analyses.

The study also demonstrated a significantly increased risk of venous thromboembolism (HR, 2.89; 95% CI, 1.50–5.58), without benefit on other cardiovascular outcomes [18]. The HERS II extension, with a total follow-up of 6.8 years, confirmed the absence of an overall cardiovascular benefit, reinforcing the conclusion that hormone therapy should not be initiated for secondary prevention [57]. Generalizability is limited by the inclusion of women with established coronary disease and a relatively high mean age (66.7 years), reflecting the initiation of therapy at an advanced stage of atherosclerotic disease. In addition, only a single therapeutic regimen was evaluated [18].

Overall, HERS demonstrated that combined hormone therapy is not effective for secondary prevention of cardiovascular disease and may be associated with increased risk during the early phase of treatment.

### 4.8. Estrogen in the Prevention of Atherosclerosis (EPAT)

EPAT was a randomized, double-blind, placebo-controlled study that evaluated the effect of oral estradiol on the progression of subclinical atherosclerosis in postmenopausal women without established cardiovascular disease. Estradiol therapy was associated with a significant slowing of CIMT progression compared with the placebo, particularly among women not receiving lipid-lowering therapy. However, the study assessed a surrogate marker CIMT and did not report clinical cardiovascular events, thereby limiting its direct relevance to cardiovascular prevention [58].

### 4.9. Women’s Estrogen for Stroke Trial (WEST Study)

The WEST Study was a randomized, double-blind, placebo-controlled trial designed to evaluate the effect of 17β-estradiol on secondary prevention of stroke in postmenopausal women with a recent history of stroke or transient ischemic attack. After a mean follow-up of approximately 2.8 years, estrogen therapy did not reduce the risk of recurrent stroke or death compared with the placebo. A secondary analysis assessed cognitive function and showed no significant effect of estradiol on global cognitive decline (RR: 0.74; 95% CI, 0.49–1.13). However, an exploratory analysis suggested a lower risk of cognitive decline among women with normal baseline cognitive function (8.8% vs. 19.6%; RR, 0.46; 95% CI, 0.24–0.87) [59].

Overall, these findings do not support a benefit of estrogen therapy in the secondary prevention of stroke or in preserving cognitive function, although they suggest a potential limited effect in women without baseline cognitive impairment.

### 4.10. Estrogen Replacement and Atherosclerosis (ERA Study)

The ERA trial was a randomized, double-blind, placebo-controlled study that evaluated the effect of hormone therapy on the progression of coronary atherosclerosis in postmenopausal women with established coronary artery disease. After a mean follow-up of 3.2 years, no significant differences were observed between the treatment and placebo groups in minimal coronary artery diameter (1.87 mm vs. 1.84 mm vs. 1.87 mm) or in the progression of atherosclerotic lesions. Although hormone therapy led to favorable changes in lipid profiles, including reductions in low-density lipoprotein cholesterol of up to 16.5% and increases in high-density lipoprotein cholesterol of up to 18.8%, these effects did not translate into structural or clinical benefits, with similar rates of cardiovascular events across groups. These findings support the lack of a protective effect of hormone therapy in the secondary prevention of coronary artery disease [60].

An overview of the most relevant clinical studies investigating the cardiovascular effects of hormone replacement therapy is presented in Table 2.

## 5. Comparative Synthesis of Evidence

### 5.1. Heterogeneity of Study Populations and Implications for Interpretation

Randomized trials evaluating hormone replacement therapy (HRT) differ substantially in the characteristics of the populations studied, contributing to variability in outcomes and limiting generalizability. In the Women’s Health Initiative (WHI), one of the largest available trials, the study population was relatively diverse, comprising approximately 75% White women, 15% African American women, and 6% Hispanic women, while other ethnic groups, including Asian populations, were underrepresented (<2%) [20,55].

In contrast, European studies such as WISDOM and DOPS included predominantly Caucasian populations, whereas smaller trials (ELITE, EPAT, KEEPS) had limited ethnic diversity and were not designed to assess differences across racial or ethnic subgroups. Although analyses from WHI did not demonstrate significant interactions between race/ethnicity and the effects of HRT on major cardiovascular outcomes [20,55], these analyses were likely underpowered.

In addition, substantial differences exist in participant age and time since menopause across studies. Secondary prevention trials (HERS, ERA, WEST) enrolled women with established cardiovascular disease or older age, whereas more recent trials (ELITE, KEEPS, DOPS) focused on younger or recently postmenopausal women. This variability further supports the “window of opportunity” hypothesis, according to which the effects of HRT are critically dependent on the timing of therapy initiation.

### 5.2. Major Cardiovascular Events (Coronary Heart Disease, Myocardial Infarction, Stroke)

In secondary prevention settings or among women many years beyond menopause, HRT has not demonstrated cardiovascular benefit. In HERS, conducted in women with established coronary artery disease, no reduction in coronary events was observed, with an early increase in risk during the first year of treatment (HR ≈ 1.5) [16]. Similar findings were reported in ERA and WEST, where no reduction in atherosclerosis progression or recurrent stroke was observed [59,60].

In the Women’s Health Initiative combined estrogen–progestin arm, hormone therapy was associated with an increased risk of coronary heart disease (HR ≈ 1.29 in the early years) and stroke (HR ≈ 1.31), particularly among older women or those initiating therapy later after menopause. In the estrogen-only WHI arm, no reduction in coronary heart disease risk was observed (HR, 0.91), while the risk of stroke was significantly increased (HR, 1.39) [20,55,56]. Similarly, in WISDOM, the risk of major cardiovascular events was increased, leading to early termination of the trial due to an unfavorable balance between risks and benefits [54]. More recently, a nationwide Swedish emulated target trial including 919,614 women aged 50–58 years reported an increased risk of ischemic heart disease among users of oral estrogen–progestin therapy (HR 1.21, 95% CI 1.00–1.46), while no significant increase in cardiovascular risk was observed with transdermal estrogen regimens, supporting the importance of treatment formulation and route of administration [50].

In contrast, studies enrolling younger or recently postmenopausal women have suggested more favorable effects. In DOPS, early initiation of therapy was associated with a reduction in a composite endpoint of mortality, myocardial infarction, and heart failure (relative reduction of approximately 50%) [51]. ELITE demonstrated a slowing of atherosclerosis progression, assessed by CIMT, only in women within 6 years of menopause [30], while EPAT showed a significant reduction in carotid atherosclerosis progression (≈0.0017 mm/year difference) [58]. In contrast, KEEPS did not demonstrate significant changes in CIMT [52,53].

### 5.3. Venous Thromboembolism and Prothrombotic Risk

Venous thromboembolism (VTE) risk represents one of the most consistent adverse effects of hormone replacement therapy (HRT), particularly with oral administration. In the Women’s Health Initiative (WHI), combined hormone therapy was associated with an approximately twofold increase in the risk of deep vein thrombosis (DVT) and pulmonary embolism (PE) (HR ≈ 2.0–2.1). In the estrogen-only arm, VTE risk was also increased (HR, 1.33), with a significant rise in DVT risk (HR, 1.47) [20,55,56].

In WISDOM, the risk of thromboembolic events was markedly elevated (HR, 7.36; 95% CI, 2.20–24.60), including both proximal DVT and pulmonary embolism [54]. These events occurred predominantly early after treatment initiation, suggesting an acute prothrombotic effect.

These effects are largely attributed to the hepatic first-pass metabolism of oral estrogens, which increases the synthesis of coagulation factors (e.g., factor VII and fibrinogen) and reduces anticoagulant proteins. In contrast, in KEEPS, the use of transdermal estradiol was associated with a neutral thrombotic profile, without significant changes in coagulation markers [53]. These observations are further supported by a large Swedish nationwide emulated target trial, which demonstrated increased venous thromboembolism risk with oral estrogen-containing regimens, whereas transdermal estrogen preparations were associated with a more favorable thrombotic profile [50].

### 5.4. Cardiovascular Risk Factors and Metabolic Profile

HRT significantly influences cardiovascular risk factors, although these effects do not consistently translate into improved clinical outcomes.

Regarding lipid metabolism, HRT is associated with reductions in low-density lipoprotein cholesterol (LDL-C) of approximately 10–15% and increases in high-density lipoprotein cholesterol (HDL-C) of 10–15%, as observed in the Women’s Health Initiative. However, oral estrogen is also associated with significant increases in triglyceride levels (up to 20–25%), with more pronounced effects compared with transdermal administration [20,55]. Despite these favorable lipid changes, no reduction in major cardiovascular events has been demonstrated, suggesting that proinflammatory and prothrombotic mechanisms may offset metabolic benefits.

With respect to glucose metabolism, HRT has been associated with a modest reduction in the incidence of type 2 diabetes (approximately 20% relative reduction in WHI), likely mediated by improved insulin sensitivity [20]. Nevertheless, this metabolic benefit has not translated into a reduction in overall cardiovascular risk.

HRT does not appear to exert a protective effect on blood pressure. In WHI, a small increase in systolic blood pressure (approximately 1 mmHg) was observed [20,55], while no significant differences were reported in WISDOM [54]. Oral estrogen may activate the renin–angiotensin system through increased hepatic angiotensinogen production, potentially contributing to these effects.

Most trials, including WHI, HERS, and KEEPS, enrolled populations with a mean body mass index (BMI) in the overweight to obese range (approximately 28–30 kg/m^2^) [18,20,55,56]. No consistent effect of MHT on body weight or fat distribution has been demonstrated, and its impact on cardiovascular risk does not appear to be mediated through changes in adiposity.

### 5.5. Heart Failure and Arrhythmias

Data regarding heart failure are limited. In DOPS, early initiation of hormone replacement therapy (HRT) was associated with a reduced risk of heart failure [51], whereas WHI and HERS did not demonstrate significant benefits [18,20,55].

With respect to arrhythmias, particularly atrial fibrillation (AF), randomized data are scarce. Observational analyses suggest an increased risk associated with current HRT use (hazard ratio [HR] ≈ 2.0–2.2), dependent on the type of preparation, with higher risk reported for conjugated equine estrogens and tibolone, and a more neutral profile for estradiol [61]. In contrast, a post hoc analysis of the AFFIRM trial did not show a significant increase in cardiovascular events among women with AF receiving HRT (HR, 0.89) [62].

### 5.6. Type of Therapy and Route of Administration

Differences between studies are also influenced by the type of hormonal preparation used. Trials reporting less favorable outcomes, such as WHI, HERS, and WISDOM, predominantly used oral conjugated equine estrogens, often in combination with medroxyprogesterone acetate [18,20,54,55,56].

In contrast, more recent studies, including ELITE, KEEPS, and DOPS, have used estradiol, in some cases administered transdermally, along with progestogens with a profile closer to endogenous hormones [30,51,53]. Transdermal administration avoids hepatic first-pass metabolism and appears to be associated with a lower thrombotic risk. These observations are supported by contemporary registry-based evidence from Sweden, which demonstrated a more favorable cardiovascular profile for transdermal estrogen regimens compared with oral estrogen–progestin therapy, particularly with regard to ischemic heart disease and venous thromboembolism risk [50].

### 5.7. Primary vs. Secondary Prevention

In secondary prevention settings (HERS, ERA, WEST), HRT has not demonstrated cardiovascular benefit and may increase the early risk of adverse events [18,59,60]. In late primary prevention (WHI, WISDOM), cardiovascular and thromboembolic risks outweigh the potential benefits [20,54,55].

In contrast, in early primary prevention (ELITE, DOPS, EPAT, KEEPS), neutral or favorable effects on subclinical atherosclerosis have been observed, with some evidence of reduced mortality in selected populations [30,51,52,53,58].

Overall, HRT does not confer a global cardiovascular benefit. The risk of myocardial infarction, stroke, and venous thromboembolism is increased, particularly with late initiation or in women with preexisting cardiovascular disease. Differences across studies support the “window of opportunity” hypothesis and underscore the need for an individualized approach based on age, time since menopause, baseline risk profile, and type of therapy used.

## 6. The “Window of Opportunity” Hypothesis

The concept of the “window of opportunity” (timing hypothesis) has become central to the interpretation of the cardiovascular effects of hormone replacement therapy (HRT). It suggests that the impact of HRT depends critically on the timing of initiation relative to menopause, as well as on the underlying vascular status of the patient.

Clinical data support the “window of opportunity” hypothesis. Secondary analyses of the Women’s Health Initiative demonstrated that women who initiated HRT within 10 years of menopause or before the age of 60 showed trends toward reduced risk of coronary heart disease, whereas late initiation (more than 20 years after menopause or after age 70) was associated with increased cardiovascular risk [55,56].

These findings have been reinforced by subsequent studies. In ELITE [58], administration of 17β-estradiol reduced the progression of atherosclerosis only in women in early postmenopause, with no benefit observed in those with long-standing menopause [48]. Similarly, the study by Schierbeck et al. (DOPS) showed that early initiation of hormone therapy was associated with reduced cardiovascular events and long-term mortality [51]. In contrast, trials conducted in older women or in those with established cardiovascular disease, such as HERS, ERA, and WISDOM, did not demonstrate benefit, and in some cases, reported increased cardiovascular and thromboembolic risk [16,53,55].

## 7. Safety Considerations and Risk Stratification

Although menopausal hormone therapy initiated within the “window of opportunity” may confer cardiovascular benefits in carefully selected women, a rigorous assessment of the safety profile remains essential. The principal risks include venous thromboembolism, stroke, and hormone-dependent malignancies, all of which must be weighed against potential benefits within an individualized risk–benefit framework.

### 7.1. Risk of Venous Thromboembolism

Oral estrogen administration is consistently associated with an increased risk of VTE, estimated at approximately twofold compared with untreated populations. This effect is primarily explained by the hepatic first-pass metabolism, which promotes a procoagulant hemostatic imbalance through the increased synthesis of coagulation factors and reduced anticoagulant activity. In absolute terms, the baseline risk is approximately 1 case per 1000 woman-years, with hormone therapy associated with an excess of about 1–2 additional events per 1000 women annually. The risk is most pronounced during the first year of treatment [63,64].

In contrast, transdermal estrogen administration appears to avoid these hepatic effects and is associated with a more favorable thrombotic profile. Observational studies have not demonstrated a significant increase in VTE risk with this route of administration [65,66]. Accordingly, in women at increased thrombotic risk—such as those with obesity, thrombophilia, immobility, or prior VTE—transdermal formulations are generally preferred.

Although the absolute risk remains low in younger women without comorbidities, it increases progressively with age and the accumulation of risk factors, underscoring the importance of careful risk assessment prior to therapy initiation.

### 7.2. Risk of Stroke

The risk of ischemic stroke in the context of hormone therapy is influenced by several factors, including the type of estrogen, dose, and route of administration. Oral therapy has been associated with a modest increase in stroke risk, particularly at higher doses. In absolute terms, this corresponds to a baseline incidence of approximately 2–3 cases per 1000 women per year and an excess of about 0.5–1 additional case per 1000 women annually (relative risk approximately 1.2–1.3) [67,68].

Lower-dose regimens and transdermal administration appear to have a more favorable cerebrovascular safety profile. In particular, low-dose transdermal estrogen (≤50 μg) has not been consistently associated with an increased risk of stroke, in contrast to higher doses, where an elevated risk has been observed [67,68].

Although the absolute risk increase is minimal in younger women without cardiovascular risk factors, it becomes clinically significant in older patients or those with comorbidities. Therefore, the optimization of modifiable risk factors—particularly hypertension—is essential prior to initiating hormone therapy.

## 8. Practical Recommendations for the Use of Hormone Therapy in Postmenopause

**Figure 1:**
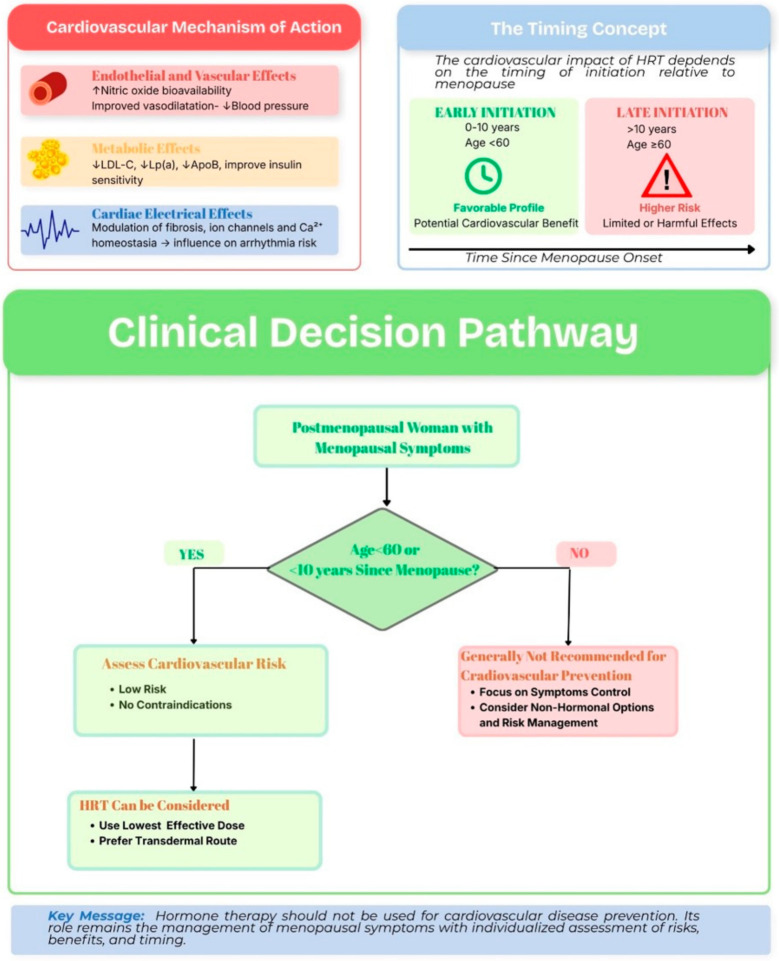
Menopausal hormone therapy: Mechanism, timing, and clinical decision-making. ↑ indicates an increase; ↓ indicates a decrease.

The main recommendations regarding the use of menopausal hormone therapy, including patient selection and timing of initiation, are summarized in Figure 1.

### 8.1. Special Considerations

#### 8.1.1. What Happens If a Woman on HRT Sustains an MI?

Available data suggest that HRT after myocardial infarction (MI) is not associated with a clear cardiovascular benefit, but neither does it appear to confer a significantly increased risk. The HERS trial did not demonstrate a reduction in coronary events in the secondary prevention setting [57]. Consistently, a nationwide Danish cohort study by Bretler et al. showed that discontinuation compared with the continuation of HRT after MI was not associated with significant differences in the risk of reinfarction or mortality [69]. More recently, another large Danish study found no significant association between post-MI HRT use and major adverse cardiovascular events (HR: 0.95; 95% CI, 0.89–1.01) [70].

In the context of the prothrombotic state following MI, the risk associated with HRT appears to depend on dose and route of administration. Estrogen exerts dose-dependent thrombotic effects, and transdermal administration is associated with a lower risk compared with oral formulations. Therefore, when HRT is considered necessary, low-dose regimens—preferably via non-oral routes—and the use of non-androgenic progestogens are recommended when indicated. Concomitant statin therapy may further reduce the risk of venous thromboembolism, with reductions of up to 55% reported [71].

#### 8.1.2. What Happens If a Woman on HRT Sustains an Acute Thromboembolism?

Current evidence and international guidelines consistently indicate that HRT is contraindicated in the presence of active VTE, including deep vein thrombosis and pulmonary embolism. In the event of an acute VTE episode in a patient receiving systemic estrogen therapy, treatment should be discontinued immediately, and standard anticoagulation should be initiated without delay. Data from observational studies and randomized trials demonstrate that VTE risk is increased during current HRT use, with higher risk associated with oral formulations and combined estrogen–progestin therapy compared with estrogen-only or transdermal regimens [67,68,69,72]. Importantly, this excess risk appears to be transient and reversible. Evidence from large cohort studies shows that former users have a VTE risk comparable to that of never-users (RR: 0.95; 95% CI, 0.84–1.08), suggesting a return to baseline risk following discontinuation of therapy [64,73,74,75]. Consistently, the elevated risk associated with current use declines rapidly after treatment cessation, without persistent excess risk among past users.

Accordingly, in patients with active VTE or a significant history of thromboembolic events, continuation or reinitiation of systemic MHT is generally not recommended, and nonhormonal alternatives should be preferred. Transdermal estrogen may be considered only in carefully selected cases, under specialist supervision and after thorough risk assessment [64,74,76].

#### 8.1.3. HRT Use in Atrial Fibrillation Under Anticoagulation

In postmenopausal women with atrial fibrillation, HRT is not an absolute contraindication but requires careful, individualized assessment of overall thromboembolic and cardiovascular risk. Data from observational studies and population-based cohorts suggest that HRT may be associated with a modest increase in the risk of AF during current use, without persistence of this effect after treatment discontinuation.

At the same time, recent large-scale analyses have reported associations between HRT use and increased incidence of both atrial and ventricular arrhythmias, as well as higher rates of hospitalization for acute heart failure, suggesting potential effects on cardiac electrical stability and hemodynamic function. In women with atrial fibrillation, current guidelines recommend thromboembolic risk stratification using validated scores and the initiation of anticoagulation when indicated, without considering HRT as a formal contraindication.

Therefore, the continuation or initiation of HRT in anticoagulated patients should be an individualized clinical decision, based on the balance between the control of menopausal symptoms and overall cardiovascular risk [61,77,78].

#### 8.1.4. What Is the Impact of HRT in Women with Heart Failure?

In heart failure, HRT remains an investigational strategy, with no robust evidence supporting benefit on major clinical endpoints, and requires strict individualization of the risk–benefit profile. Available data support the concept of an “anabolic hormonal deficiency” in chronic heart failure. Small clinical studies have suggested potential improvements in exercise capacity, left ventricular function, and quality of life following selective hormonal supplementation; however, these findings have not been consistently confirmed in larger trials. At the same time, some evidence indicates potential adverse effects, including fluid retention, proarrhythmic effects, and increased thromboembolic risk depending on the type of hormonal intervention and patient profile. These factors may contribute to clinical decompensation or worsening of disease status in vulnerable patients [40,79,80,81,82].

At present, HRT in heart failure should be considered as an investigational approach, reserved for carefully selected patients with documented hormonal deficiencies and managed within a multidisciplinary framework. Large-scale randomized controlled trials are needed to better define its long-term efficacy and safety profile.

## 9. Limitations

This review has several limitations. First, it was a narrative review rather than a systematic analysis, which inherently introduces a risk of selection bias. Second, the included studies were highly heterogeneous with respect to study populations, timing of therapy initiation, types of hormonal preparations, routes of administration, and evaluated endpoints. In addition, a substantial proportion of the evidence derived from studies that used surrogate markers of atherosclerosis, which do not necessarily translate into reductions in major cardiovascular events. Furthermore, many of the trials reporting unfavorable outcomes evaluated traditional oral hormonal regimens in older women, limiting the direct applicability of these findings to contemporary formulations and to the early initiation of therapy in clinical practice.

## 10. Conclusions

Menopausal hormone therapy is not indicated for the prevention of cardiovascular disease, as clinical trials have demonstrated a lack of overall cardiovascular benefit and an increased risk of thromboembolic and cerebrovascular events in certain clinical contexts.

The cardiovascular effects of HRT are influenced by the timing of initiation, type of preparation, and route of administration, with more favorable profiles observed with early initiation and the use of transdermal estrogen and more physiologic progestogens. However, consistent evidence supporting a reduction in major cardiovascular events is lacking. Based on current evidence, MHT should be prescribed following an individualized risk–benefit assessment, primarily for the management of menopausal symptoms and the prevention of osteoporosis, rather than for cardiovascular disease prevention.

## Figures and Tables

**Table 1:** Recommendations for hormone therapy from different medical societies.

Aspect of Treatment	European Society of Endocrinology [2]	American Association of Clinical Endocrinology and American College of Endocrinology [46]	North American Menopause Society [47]	American College of Obstetricians and Gynecologists [48]	European Menopause and Andropause Society [49]
**Principal indication**	Menopausal symptoms; prevention of bone loss; mandatory use in premature ovarian insufficiency	Menopause symptoms	Menopause symptoms, particularly vasomotor symptoms	Menopause symptoms	Menopause symptoms and management of long-term health risks
**Prevention of coronary heart disease**	Not recommended as primary or secondary prevention of cardiovascular disease	Not recommended	Not recommended	Not recommended	Not recommended as a primary indication; potential benefits depend on age and timing of initiation
**Special considerations**	Individualized assessment including age, time since menopause (<10 years), cardiovascular and cancer risk profile; shared decision-making; consideration of comorbidities	Consideration of age, time from menopause, and cardiovascular risk factors; individualized use in diabetes; micronized progesterone preferred; bioidentical hormones not recommended	Consideration of age, time since menopause onset, cardiovascular risk, and contraindications; hormone therapy should be considered in women younger than 60 years and within 10 years of the final menstrual period, if no contraindications are present	None	Consideration of age, time since menopause onset, individual cardiovascular and cancer risk profile; particular attention to premature ovarian insufficiency
**Dose and route of administration**	Individualized; preference for transdermal estrogen in women with cardiovascular risk factors; choice based on patient characteristics and preferences	Individualized; transdermal estrogen may be preferred over oral preparations	Appropriate dose to manage symptoms, with consideration of route	Lowest effective dose	Individualized; multiple routes available (oral, transdermal, vaginal), chosen based on patient characteristics and risk profile
**Duration of use**	No fixed limit; use for the shortest duration consistent with treatment goals, with periodic reassessment; continuation possible beyond 60 years based on individualized risk–benefit evaluation	Individualized according to risk–benefit assessment	May be extended for persistent vasomotor symptoms, prevention of bone loss, or quality of life, with regular reassessment of benefits and risks	Shortest period based on risk-benefit analysis, with recommendation against routine discontinuation in patient ≥ 65 y of age	Typically around 5 years for vasomotor symptoms; may be continued longer based on individual risk-benefit assessment; recommended until natural age of menopause in premature ovarian insufficiency

**Table 2:** Relevant clinical studies regarding the cardiovascular effects of HRT.

Trial	Participants	Population (Mean Age/Timing)	Intervention	Follow-Up	Prevention Type	Main Cardiovascular Findings
**Swedish Nationwide Trial (2024)**	919,614	Women aged 50–58 years	Oral or transdermal estradiol-based menopausal hormone therapy with or without a progestogen, oral estrogen plus LNG-IUS, or tibolone	Median 8 years	Primary	Oral estrogen–progestin associated with ↑ ischemic heart disease and VTE; transdermal estrogen showed a more favorable cardiovascular profile
**ELITE (2016)**	643	Early (<6 years postmenopause; ~55 y) vs. late (≥10 years; ~65 y)	Oral estradiol ± progesterone	Median 5 years	Primary	Slower progression of CIMT in early group; no benefit in late initiation; no reduction in clinical events
**DOPS (2012)**	1006	Recently postmenopausal women (~50 y)	Estradiol ± norethisterone acetate	10 years + 16 years follow-up	Primary	Reduced mortality, MI, HF; no ↑ stroke/VTE; benefit only with early initiation
**KEEPS (2012)**	727	Recently postmenopausal (~52 y)	Oral/transdermal estradiol + progesterone	4 years	Primary	Neutral on IMT and coronary calcium; no ↑ CV events
**WISDOM (2007)**	~5600	Postmenopausal (~63 y; late)	CEE + MPA	~1 year	Primary	↑ coronary + thromboembolic events; VTE HR ~7.36
**WHI—Estrogen alone (2004)**	10,739	Postmenopausal with hysterectomy (~63 y)	CEE 0.625 mg	6.8 years	Primary	No ↑ CHD; ↑ stroke; possible ↓ MI in younger subgroup
**WHI—E + P (2002)**	16,608	Postmenopausal 50–79 y (~63 y; late)	CEE + MPA	5.2 years	Primary	↑ CHD, stroke, VTE; early excess coronary risk
**HERS II (2002)**	2763	Women with CHD	CEE + MPA	6.8 years total	Secondary	No cardiovascular benefit
**EPAT (2001)**	222	Healthy postmenopausal (~55 y)	Oral estradiol 1 mg	2 years	Primary	↓ progression of CIMT (surrogate only)
**WEST (2001)**	664	Prior stroke/TIA (~71 y)	Estradiol	2.8 years	Secondary	No reduction in stroke or mortality
**ERA (2000)**	309	CAD (~65 y)	CEE ± progestin	~3 years	Secondary	No effect on atherosclerosis progression
**HERS (1998)**	2763	Established CHD (~67 y)	CEE + MPA	4.1 years	Secondary	No benefit; early ↑ events; ↑ VTE

VTE—venous thromboembolism; LNG-IUS—levonorgestrel-releasing intrauterine system; CEE—conjugated equine estrogens; MPA—medroxyprogesterone acetate; CIMT—carotid intima-media thickness; CHD—coronary heart disease; MI—myocardial infarction, TIA—transient ischemic attacks; ↑ indicates an increase, ↓ indicates a decrease.

## Data Availability

No new data were created or analyzed in this study.
